# Assessment of the available evidence for the use of 7‐Tesla (T) magnetic resonance imaging (MRI) in neurological and musculoskeletal disorders, with comparison to 3‐T and 1.5‐T MRI: A systematic scoping review

**DOI:** 10.1111/ene.16557

**Published:** 2024-12-15

**Authors:** Piotr Radojewski, Gian Franco Piredda, Gabriele Bonanno, Karl‐Olof Lövblad, Maria Isabel Vargas, Reto Sutter, Daniel Nanz, Tanya Karrer, Georgia Salanti, Roland Wiest

**Affiliations:** ^1^ University Institute of Diagnostic and Interventional Neuroradiology Inselspital, Bern University Hospital, University of Bern Bern Switzerland; ^2^ Translational Imaging Center (TIC) Swiss Institute for Translational and Entrepreneurial Medicine Bern Switzerland; ^3^ Advanced Clinical Imaging Technology Siemens Healthineers International AG Lausanne Switzerland; ^4^ CIBM Centre for Biomedical Imaging Geneva Switzerland; ^5^ Division of Diagnostic and Interventional Neuroradiology Geneva University Hospital Geneva Switzerland; ^6^ Service de Radiologie Clinique des Grangettes‐Hirslanden et Faculté de Médecine de l'Université de Genève Switzerland; ^7^ Radiology Department Balgrist University Hospital, University of Zurich Zurich Switzerland; ^8^ Swiss Center for Musculoskeletal Imaging Zurich Switzerland; ^9^ University Library University of Bern Bern Switzerland; ^10^ Institute of Social and Preventive Medicine University of Bern Bern Switzerland

**Keywords:** 7‐T MRI, epilepsy, evidence, MS, neuroimaging, UHF

## Abstract

**Background:**

Ultra‐high‐field magnetic resonance imaging (MRI) at a field strength of 7 Tesla (T) has marked a significant milestone in diagnostic imaging since it was approved for clinical use in 2017. Despite the potential to improve image analysis by advances in signal‐to‐noise ratio, and improved spatial resolution and metabolic imaging, the clinical implementation of 7‐T MRI remains limited. Factors that contribute to this limited availability are the high price, the operating costs, the need for specifically educated personnel, and lack of evidence of clinical benefit.

**Methods:**

The aim of this scoping review was to evaluate the evidence of the clinical advantages of 7‐T MRI versus MRI at lower field strengths, complementary imaging modalities, and diagnostic standard approaches for neurological and musculoskeletal disorders. We searched MEDLINE, CENTRAL, Embase and Web of Science for this review.

**Results:**

We identified 1966 studies, of which 83 were included in our review. Most studies (73 studies, 88%) examined neurological indications, nine studies (11%) examined musculoskeletal indications, and one study reported on peripheral arterial occlusive disease. Of the neuroimaging indications, cerebrovascular diseases were the most frequently investigated (14 studies), followed by multiple sclerosis (13 studies) and epilepsy (11 studies).

**Conclusion:**

The available comparative evidence varied greatly across indications, with the best‐documented evidence being for imaging of epilepsy. Risk of bias overall was high, with limitations in blinding information, study design reporting, and patient recruitment details. The identified evidence gaps underscore the need for comparative research to determine appropriate indications and to understand whether the potential diagnostic advantage of 7‐T MRI translates to a tangible clinical benefit for patients. Future studies should include clinically relevant patient outcomes that go beyond radiological metrics.

## INTRODUCTION

The first ultra‐high‐field magnetic resonance scanner was approved for clinical use in 2017, receiving the CE Mark and US Food and Drug Administration 510(k) clearance for imaging of the head and knee [1, 2]. As a result, 7‐Tesla (T) magnetic resonance imaging (MRI) examinations can be performed based on clinical indication . Technical advances in this imaging method encompass a high signal‐to‐noise ratio, high resolution, and high contrast for improved depiction of abnormalities. Technical limitations of the ultra‐high‐field technique include increased magnetic field inhomogeneity (B0 and B1), with increased imaging artifacts and increased specific absorption rate.

While 7‐T MRI is a powerful imaging tool, the clinical availability of 7‐T MRI is still limited [3]. Factors that contribute to the limited availability of this promising technique might be the higher price, higher operating costs, the need for specifically educated personnel, and lack of evidence of a clinical benefit compared to standard imaging techniques. Another less frequently discussed factor potentially contributing to the limited clinical use of 7‐T MRI is the fact that many of these systems are installed in research centers and not in hospitals. Due to the broad availability of conventional 1.5‐T and 3‐T MRI systems, these scanners will likely remain the standard imaging tools for neurological disorders, whereas 7‐T MRI could be seen as a complementary tool to solve challenging and unanswered clinical questions that have not been solved by routine MRI [4, 5, 6]. Since the signal‐to‐noise ratio increases with field strength, the most evident application at 7 T is to gain spatial resolution. Seven‐Tesla MRI has been postulated to supply added morphological, functional, metabolic, and biochemical information. Within the neuro‐research context, 7‐T MRI has been widely implemented, for example, in the imaging of multiple sclerosis (MS), cerebrovascular diseases, epilepsy, brain tumors and neurodegenerative disorders [5, 7]. A significant number of studies have been published in the field of 7‐T MRI, establishing new methods and laying the necessary foundation for potential clinical applications of such systems [8]. However, several years after its approval, the added diagnostic value of 7‐T MRI, with focus on direct comparison with lower field strengths and other imaging modalities, is unclear, with only few indications such as epilepsy becoming established [3, 9]. Only few systematic reviews for selected conditions or techniques are available, for example, for epilepsy or vessel wall imaging [10, 11]. This starting point is comparable to that of 3‐T imaging more than a decade ago. In a systematic review comparing 1.5‐ and 3‐T MRI in 2012, Wardlaw et al. stated that “objective evidence to guide MRI purchasing decisions and routine diagnostic use (*of 3T MRI*) is lacking” [12]. These authors concluded that “rigorous evaluation accuracy and practicalities of diagnostic imaging technologies should be the routine, as for pharmacological interventions, to improve effectiveness of healthcare.” With increasing economic pressure on healthcare systems, this is even more relevant today. More than a decade later, 3‐T MRI is an accepted and well‐established diagnostic method, with proven added value. Notably, the evidence accumulated gradually over decades after its approval for clinical use. The aim of this study was to perform a scoping review to provide information on the comparative evidence for the clinical use of 7‐T MRI, and to identify potential gaps in evidence to trigger further research. Specific focus was placed on the search for clinical value and outcomes that are directly meaningful for patients.

## METHODS

### Inclusion criteria

We included studies examining patients with 7‐T MRI compared with other diagnostic methods, mainly lower‐field‐strength MRI or other techniques, when suitable for the indication. We included all neuro‐ and musculoskeletal indications as defined by the availability of clinically approved coils. There were no further specific exclusion criteria related to patients or diseases. Reasons for exclusion were selected based on pre‐specified categories (background articles, foreign language publications, inappropriate outcome, inappropriate population, inappropriate publication type, inappropriate study design, inappropriate study duration, studies using phantom or animal models).

### Study identification

To identify all potentially relevant studies, we designed literature searches for the following information sources: MEDLINE (Ovid), Ovid MEDLINE(R) ALL, Embase (Ovid), Cochrane Library CENTRAL (Wiley), and Web of Science (Clarivate), with the last search conducted in May 2023. An initial search strategy was developed in MEDLINE by a medical information specialist and was tested against a list of core references to see if they were included in the search result. No limits were applied in any database regarding study type, language, publication year or any other formal criteria. Details and the exact search strategies are presented in Appendix S1. In addition to the electronic database searches, reference lists and bibliographies from relevant publications were checked for relevant studies. All identified citations were deduplicated using the deduplication tool Deduklick [13] and according to the Bramer method [14]. The screening of titles and abstracts was performed by two reviewers and tested against the inclusion criteria.

The results of the searches were imported into Rayyan software [15]. Titles and abstracts of all the identified studies were independently examined by two reviewers and classified as eligible or ineligible. Records automatically marked by the software for potential exclusion (for example, due to being a phantom‐based or animal study) were manually checked for confirmation. Subsequently, two reviewers examined the included full texts.

### Data extraction and analysis

Two reviewers (P.R. and G.F.P.) independently extracted data and evaluated the quality of the included studies using the revised tool for the quality assessment of diagnostic accuracy studies, QUADAS‐2 [16]. Consensus was reached by discussion between the reviewers in case of discrepancies.

From the included studies, type of publication (journal article or conference abstract), field (e.g., neuroimaging, musculoskeletal imaging), disease, disease subtype, study design, number of study arms, sample size and comparator imaging techniques were extracted. If a diagnostic criterion was used for the evaluation of the performance of the imaging techniques, this was noted. We aimed to synthesize the diagnostic accuracy of 7‐T MRI versus any comparator, as estimated in the included studies using standard meta‐analytical techniques (Cochrane Handbook Chapter 10: Analysing data and undertaking meta‐analyses [17]).

## RESULTS

**FIGURE 1:**
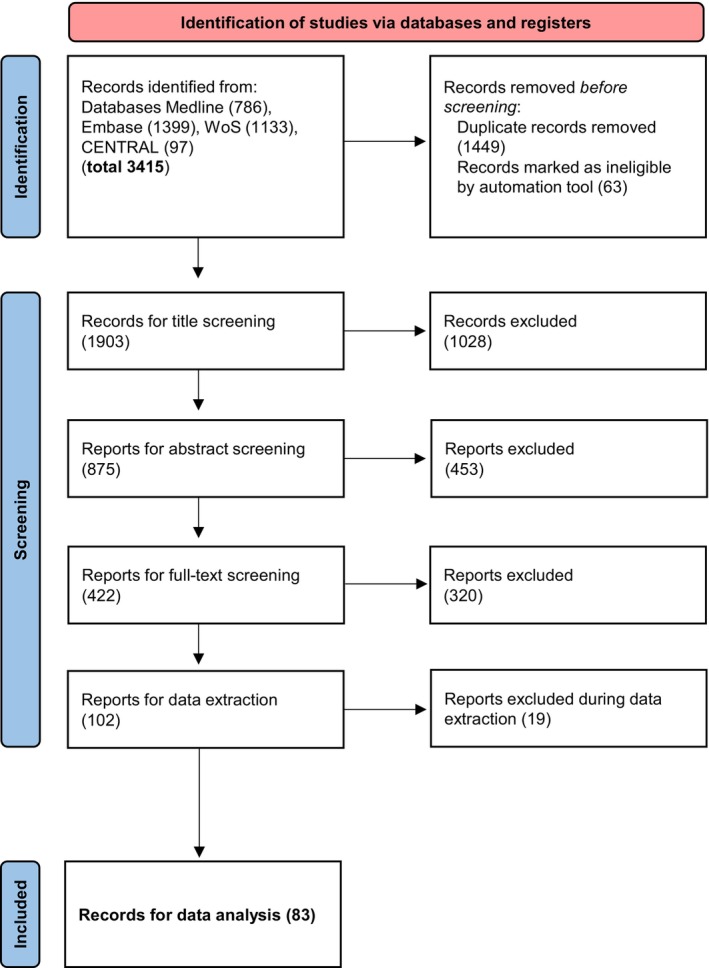
Flow chart showing the selection of studies.

We identified 3415 studies during the search. After duplicate removal and initial screening steps, 422 studies were included in the full‐text screening and 102 studies remained for data extraction. During the data extraction process, a further 19 studies were excluded (missing comparator, no inclusion of patients, no original data). Reasons for exclusion were selected based on the pre‐specified categories in the software tools and can be summarized in four categories: (i) inappropriate population studied (mainly due to inclusion of healthy individuals without inclusion of patients or study of a disease); (ii) inappropriate study design (mainly studies examining 7‐T MRI only, without comparison to other imaging techniques, modalities or magnetic resonance field strengths); (iii) inappropriate publication type (mainly preclinical studies with phantom measurement or animal trials); and (iv) background article type (no original data reported by the article). Finally, 83 studies remained for analysis (Figure 1, Appendix S2).

Of the 83 studies, 73 were journal articles, with the remaining studies comprising congress abstracts and presentations. No randomized controlled trials were identified. The most frequent study design was cross‐sectional (73 studies). In addition, we identified five longitudinal studies, four case series and one case–control study. The comparators were 3‐T MRI in 47 studies (57%) and 1.5‐T MRI in eight studies (10%). Nine studies (11%) used multiple comparators including 3‐T MRI (in conjunction with computed tomography [CT], positron emission tomography [PET], arthroscopy or bone mineral density). The remaining studies used other comparators including CT, digital subtraction angiography (DSA), PET, single‐photon emission CT, ultrasonography, and unspecified lower‐field MRI, or a combination thereof. Five studies fulfilled the criteria for diagnostic test accuracy [6, 18, 19, 20, 21]; the majority of studies did not fulfill the formal criteria for diagnostic test accuracy.

**FIGURE 2:**
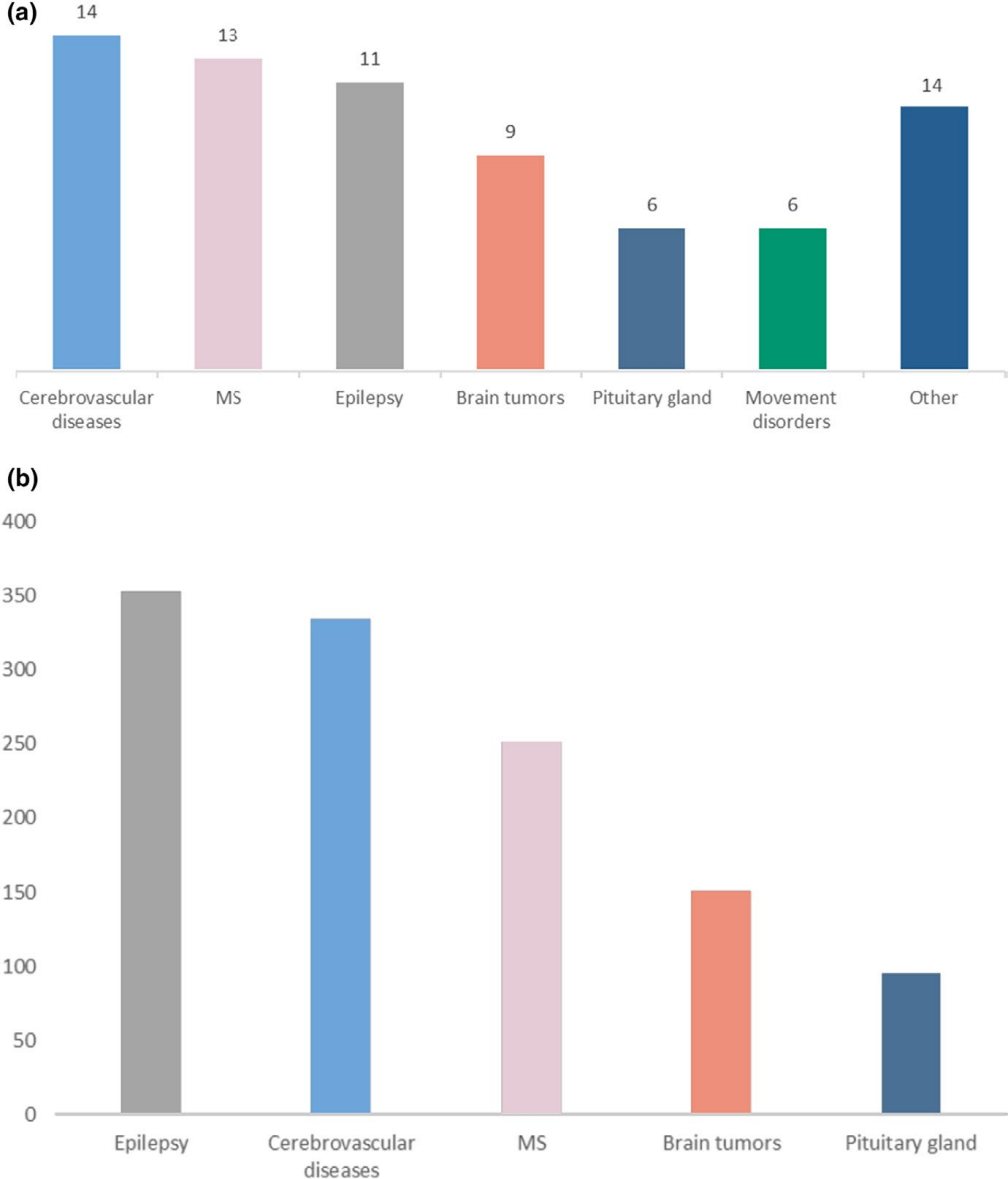
(a) Number of studies by indication for 7‐Tesla magnetic resonance imaging within the neuroimaging field. (b) Total number of included patients for five major indications for neuro‐imaging. MS, multiple sclerosis.

The majority of studies covered the field of neuroimaging (73 studies, 88%). Nine studies (11%) examined musculoskeletal disorders and one study reported on peripheral arterial occlusive disease. Among neuroimaging indications, cerebrovascular diseases were the most frequently investigated (14 studies, 19%), followed by MS (13 studies, 18%) and epilepsy (11 studies, 15%; Figure 2a). Other indications comprised neuro‐oncology (9 studies, 12%), disorders of the pituitary gland (6 studies, 8%) and movement disorders (6 studies, 8%). The remaining studies were labeled as “other indication” (14 studies, 19%) and included studies reporting on multiple disorders (two studies), amyotrophic lateral sclerosis (one study), traumatic brain injury (two studies), neuroopthalmology (two studies) depression (two studies), forensic indications (craniocerebral gunshots, one study), hearing loss (one study), head and neck cancer (one study) and functional MRI in presurgical setting (one study).

In the musculoskeletal group, the indications were knee joint cartilage defects (three studies), osteoarthritis (two studies), osteoporosis (one study), knee pain (one study), chondrocalcinosis (one study), peripheral arterial occlusive disease (one study), and orofacial reconstruction with peroneal flap (one study).

The sample size of the studies varied from three to 113 patients (median 16 patients). The cumulative number of patients examined in all studies was 1845 (with varying levels of patients included in final analyses and subgroup analyses). Epilepsy studies included the largest cumulative number of patients (353) followed by cerebrovascular indications (334) and MS (251). Neuro‐oncological studies included 151 patients and studies on pituitary gland disorders included 95 patients (Figure 2b). Cerebrovascular diseases could be subdivided into four subgroups: aneurysms (four studies), stroke/large vessel occlusion (four studies), malformations other than aneurysms including arteriovenous malformation (three studies), and small vessel disease (three studies).

The most common indication in the epilepsy group was investigation of refractory epilepsy in patients with non‐lesional epilepsy classified by standard imaging or in a pre‐surgical setting (8/11 studies). In six studies the comparator was 3‐T MRI, in two studies it was 1.5‐ or 3‐T MRI, in two studies it was 1.5‐T MRI, and in one study it was 1.5‐ and 3‐T MRI. All studies were classified as cohort studies. Six studies examined detection rates and the diagnostic yield in general, one study had the primary goal of assessing surgery outcomes, two studies examined abnormalities in the mesial temporal lobe (hippocampus sclerosis, hippocampal architecture), one study focused on workflow and protocol comparisons, and one study examined specifically the added value of 7‐T MRI in cases with previously visible lesions. Six studies reported surgery outcomes as any type of outcome (primary, secondary, etc.) for the entire study cohort or a subgroup, four studies specifically reported Engel score, two studies reported correlation with histopathology, and one study reported change in diagnosis . All studies reported on lesion detection, lesion conspicuity, contouring of lesions or a variation of such outcomes. Inter‐rater and intra‐rater (between field strengths) agreements were reported in two studies.

The studies examining MS reported lesion detection and lesion characterization (13 studies). Three‐Tesla was the comparator in 10 studies, while the comparator was 1.5‐ and 3‐T MRI in one study, 1.5‐ or 3‐T MRI in one study and an unspecified lower field strength in one study. There were 11 cross‐sectional cohort studies, one longitudinal study, and one case series. The type of MS was not specified, not reported or unknown in six studies, three studies specifically examined relapsing‐remitting MS and two studies examined various MS subtypes. One study examined relapsing‐remitting MS and secondary progressive MS, while three studies included healthy individuals for comparison. Four studies focused primarily on lesion detection in general. Three studies focused on exploring the central vein sign. Two studies focused specifically on the detection of cortical lesions. One study focused on characterization of cerebellar pathologies. One study focused on meningeal enhancement. One study focused on the detection of paramagnetic rim lesions. One study reported on the characterization of structural dynamics in MS. Nine studies reported lesion number/lesion count. Two studies reported correlation with clinical score. Three studies reported inter‐ and intra‐rater reliability. No treatment changes or patient outcomes were reported.

Among the nine studies examining applications of 7‐T MRI in neuro‐oncology, two studies were longitudinal and the remaining were cross‐sectional cohort studies. Three‐Tesla MRI was the comparator in seven studies, 3‐T MRI and amino acid PET were the comparators in one study and amino acid PET alone was the comparator in one study. All studies reported heterogeneous correlations of various imaging findings. Eight studies reported on glial tumors and one included various brain tumors (including meningioma, metastases and other entities). Of these eight studies, two studies examined grade IV tumors (according to pre cIMPACT‐NOW update) and the remaining studies examined patients with tumors of various grades, with grades not specified in three studies. No patient outcomes or resulting treatment changes were reported [22].

Among the six studies on the pituitary gland, the clinical question addressed was adenoma localization. Histopathology correlation or correlation with surgical findings was assessed in five studies. Surgical or endocrinological outcomes were provided in three studies. Formal diagnostic test accuracy was available in three studies. In one study, the comparator was 3‐T MRI, in one study it was 1.5‐T MRI, in three studies it was 1.5‐T or 3‐T MRI, and in one study it was an unspecified field strength.

Studies on movement disorders examined the use of 7‐T MRI for initial diagnosis (three studies) and deep brain stimulation planning (three studies).

Studies on cerebrovascular diseases were heterogeneous and could be divided into four subgroups: aneurysms (four studies), stroke/large vessel occlusion or stenosis (four studies), malformations other than aneurysms including arteriovenous malformation (three studies), and small vessel disease (three studies). The comparator was 3‐T MRI in six studies, 1.5‐T MRI in three studies, DSA in one study, both DSA and lower‐field‐strength MRI in two studies, and 1.5‐T or 3‐T MRI in one study. No longitudinal studies were identified. The studies reported outcomes specific to the disease, which can be summarized as lesion detection or characterization.

In the musculoskeletal field, of the nine studies, seven focused on various aspects of knee joint imaging and one on general bone health and osteoporosis. Indications for imaging of the knee joint encompassed osteoarthritis, pain, cartilage lesions, and meniscal damage. One study reported on the assessment of perforating arteries for the planning on orofacial reconstructive surgery. Seven studies were cross‐sectional and two were longitudinal. The comparator was 3‐T MRI in all but one study (which used CT angiography). Two studies used a combined comparator of 3‐T MRI with CT or arthroscopy (study on meniscal damage).

The risk of bias and quality assessment of the included publications was performed using items on the QUADAS‐2 tool (Table 1). The quality of studies varied greatly. No randomized controlled trials and a relatively low number of prospective studies were identified. The reporting of study design was lacking general information in many cases, being either not reported in 34 studies (41%) or unclear in two studies (2.4%). A detailed description of patient recruitment was lacking in a large number of studies (24, 29%). Time between the acquisition of 7‐T MRI and the comparator diagnostic examination was often not reported, or was unclear or unknown (total 43, 52%). Reports on drug treatments or changes of treatment between the examinations were often not included. Blinding of readers was not reported in 20 studies (24%) and unclear in 12 studies (14.5%). No blinding was reported in 14 studies (17%). We encountered major difficulties in assessing the study blinding status during the data extraction process, indicating a lack of transparency in the literature. The depth of technical description of the imaging methods varied greatly, possibly limiting the reproducibility of several studies. The high level of study heterogeneity, varying quality, and the fact that the diagnostic test accuracy paradigm criteria were met at least partially only for five studies did not allow a meta‐analysis.

**TABLE 1:** Items on the revised quality assessment tool for diagnostic accuracy studies, QUADAS‐2.

QUADAS‐2 item	Yes	No	Unclear	Not reported
Prospective study design	39	8	2	34
Relevant to disease spectrum	77	0	5	1
Subject recruitment clear (inclusion/exclusion adequately reported)	59	24	0	0
Time between acquisitions short enough	37	4	5	37
Treatment performed between acquisitions	2	57	1	23
Same subjects at both imaging modalities	83	0	0	0
Imaging described in replicable detail	47	23	13	0
Blinding of readers to alternative imaging modality	38	13	12	20
Data on observer reliability/reproducibility	32	51	0	0
Reporting of withdrawals	73	4	6	0
Reporting of image artifacts	42	41	0	0

## DISCUSSION

Although 7‐T MRI was approved as a diagnostic tool in 2017, a comprehensive review of the available comparative evidence was not available at the time of writing. Our aim was to collect, assess and synthesize the evidence of the diagnostic features of 7‐T MRI compared to the standard of care, usually lower‐field‐strength MRI or other imaging methods. This scoping review provides a broad overview of the evidence for potential improvement in diagnostic procedures compared to the standard of care.

Our study identified two major gaps in the evidence caused by the limited number and quality of the studies. First, the amount of comparative evidence varies substantially among the indications. While for epilepsy, as reflected in the current consensus [9], and to a lesser degree for pituitary gland pathologies, the benefit of 7‐T MRI is well documented and the indications well defined, clear evidence of benefit is not available for MS or neuro‐oncology indications. A small, but relatively robust body of evidence is available for the examination of the knee joint, mostly focused on assessment of the menisci and articular cartilage. Second, the quality of studies varies greatly, as demonstrated by the QUADAS‐2 analysis, with the majority of studies having a retrospective study design. Hard outcomes related to patient survival, therapeutic efficacy, quality of life or similar measures are rarely reported. These outcomes are best documented for imaging of epilepsy and are not available for the majority of other reported indications. Change in clinical management or in efficacy of diagnostic thinking or impact on the diagnostic process as a whole was rarely examined and was mainly available in epilepsy, pituitary gland studies and aneurysm detection. The main reported outcomes were strictly related to radiology, such as lesion count, conspicuity, appearance, diagnostic yield in absolute numbers, diagnostic confidence, intra‐ and inter‐rater agreement, and image quality. While important, such outcomes only indirectly translate into patient benefit.

Appropriate indications for the use of 7‐T MRI need to be clearly defined and backed with evidence. The first attempts have been made recently; in epilepsy imaging, 7‐T MRI promises a higher detection rate of structural lesions [9, 11, 23], which can turn patients deemed not eligible for therapeutic surgery into eligible patients. In neuro‐oncology, molecular characterization for tumor grading is a potential advantage of 7‐T MRI [24]. For diagnosing MS, the number of detected lesions, in particular cortical lesions, can be increased [25], potentially translating into patient benefit through securing a diagnosis in ambiguous cases at symptom onset. Finally, vessel wall enhancement of contrast agent can be measured in arteriosclerosis and unruptured intracranial aneurysms [10], yet the clinical benefit remains to be defined.

The available comparative evidence for the superiority of ultra‐high‐field MRI is heterogeneous and varies greatly between indications, with the largest body of evidence available in epilepsy imaging. Evidence‐based medicine is the paradigm both for an individual doctor's decisions and for global healthcare policies. Our study findings therefore have implications for research and the clinical use of 7‐T MRI; the generation of dedicated comparative evidence is essential for a broader definition of indications for 7‐T MRI with clinically relevant patient benefit. Such studies should take into account endpoints relevant for patients or socioeconomic factors, in addition to the classical radiological outcome measures.

## AUTHOR CONTRIBUTIONS

**Piotr Radojewski:** Conceptualization; investigation; writing – original draft; validation; visualization; formal analysis; data curation; writing – review and editing. **Gian Franco Piredda:** Conceptualization; investigation; writing – original draft; writing – review and editing; validation; visualization; formal analysis; data curation. **Gabriele Bonanno:** Data curation; writing – review and editing; investigation. **K.O. Lovblad:** Writing – review and editing; investigation; data curation. **Maria Isabel Vargas:** Investigation; data curation; writing – review and editing. **Reto Sutter:** Writing – review and editing; conceptualization; investigation; data curation; validation. **Daniel Nanz:** Investigation; data curation; writing – review and editing. **Tanya Karrer:** Writing – original draft; writing – review and editing; investigation; methodology; software; formal analysis; validation. **Georgia Salanti:** Resources; supervision; formal analysis; conceptualization; methodology; visualization; writing – review and editing; validation. **Roland Wiest:** Writing – original draft; conceptualization; investigation; writing – review and editing; formal analysis; data curation; supervision; resources; validation.

## CONFLICT OF INTEREST STATEMENT

The authors G.F.P. and G.B. are employed by Siemens Healthineers Intl. AG, Switzerland.

## Supporting information


Appendix S1.

Appendix S2.

## Data Availability

The data that support the findings of this study are available in the supplementary material of this article.
