# Supplementary material for: Assessment of the available evidence for the use of 7‐Tesla (T) magnetic resonance imaging (MRI) in neurological and musculoskeletal disorders, with comparison to 3‐T and 1.5‐T MRI: A systematic scoping review
Source: Eur J Neurol. 2024 Dec 15;32(1):e16557. doi: 10.1111/ene.16557 (PMC11647057; doi:10.1111/ene.16557)
Supplement: Supplementary file 1 — Appendix S1. [file ENE-32-e16557-s001.docx]

**Appendix 1**

**Detailed search strategy**

To identify potentially relevant publications on the topic, a search strategy was designed, and investigated in MEDLINE, Embase, Cochrane CENTRAL, and Web of Science. The core concepts of the search strategy consisted of 1. 7 Tesla imaging, and 2. possible patient outcomes. A medical information specialist developed an initial search strategy in Medline and tested it against a list of core references to ensure key publications were included. After refinement, the information specialist set up the search strategy for each information source based on database-specific index terms and free text. The free text search included synonyms, acronyms, and similar terms. No database-provided limits have been applied in any sources considering study types, languages or any other formal criteria. Studies concerning exclusively animals were excluded from the searches by using a double-negative search strategy based on the "Humans only" filters by Ovid. The study types meta-analyses, systematic reviews and case reports were excluded via the database field publication type (pt) and title (ti). The search was first run on 16/02/2022. An update search was performed and finalized on 02/05/2023. The results were deduplicated using the automated deduplication tool deduklick (<https://www.risklick.ch/products/deduklick/>)^[[1]](#footnote-1)^.

| **Search date** | **Database searched** | **Platform** | **Years of coverage** | **Records** |
| --- | --- | --- | --- | --- |
| 02 May 2023 | Medline | Ovid | 1946 – 2023 May 1 | 786 |
| 02 May 2023 | Embase | Ovid | 1974 - 2023 May 1 | 1399 |
| 02 May 2023 | Cochrane Central Register of Controlled Trials | Wiley | 1992 - present | 97 |
| 02 May 2023 | Web of Science Core Collection | Clarivate | 1900 - present | 1133 |
|  |  |  | Sum of references | 3415 |
|  |  |  | Removed duplicates | - 1512 |
|  |  |  | **Total references** | **1903** |

**Database Searches**

********************************************

**Ovid MEDLINE(R) ALL <1946 to May 01, 2023>**Search date: 2023/05/02

1 ("7T" or "ultra high field" or uhf or "7 T" or "7 Tesla" or "seven tesla" or "7.0T" or "7.0Tesla" or "7.0 T" or "7.0 Tesla").ti,ab,kf. 9733

2 (imaging or mri).ti,ab,kf. or exp magnetic resonance imaging/ 1325386

3 1 and 2 6094

4 ("clinical outcome" or "clinical outcomes" or expenditure* or expens* or benefi* or cost$2 or utilit* or assessment* or satisfaction or satisfied or effectiv* or success or survival* or recurrence* or progression or (quality adj2 life) or (therap* adj2 decision*) or "diagnostic accuracy" or precis* or impact or economic* or performance or financ*).ti,ab,kf. 9276636

5 ec.fs. 442579

6 exp treatment outcome/ or exp outcome assessment health care/ or exp patient satisfaction/ 1401760

7 4 or 5 or 6 10028343

8 exp Patients/ or (patient* or diseas* or client* or injur* or disorder* or ill* or sick*).ti,ab,kf. 12216385

9 3 and 7 and 8 1108

10 (exp animals/ or animal experimentation/ or models, animal/ or exp plants/ or exp fungi/) not humans/ 5543246

11 ((animal or animals or canine* or dog or dogs or feline or hamster* or lamb or lambs or mice or monkey or monkeys or mouse or murine or pig or pigs or piglet* or porcine or primate* or rabbit* or rats or rat or rodent* or sheep* or veterinar*) not (human* or patient*)).ti,kf,jw. 2535275

12 10 or 11 5988729

13 9 not 12 895

14 (meta analysis or review or systematic review or case report).pt. or ("meta analysis" or "metaanalysis" or review or "case report" or "case study" or "case studies").ti. 3791393

15 13 not 14 786

********************************************

**Embase <1974 to 2023 May 01>**Search date: 2023/05/02

1 exp nuclear magnetic resonance imaging/ 1221236

2 (imaging or mri).ti,ab,kf. 1731088

3 1 or 2 2144197

4 ("7T" or "ultra high field" or uhf or "7 T" or "7 Tesla" or "seven tesla" or "7.0T" or "7.0Tesla" or "7.0 T" or "7.0 Tesla").ti,ab,kf. 13692

5 3 and 4 9248

6 exp treatment outcome/ 2229656

7 exp outcome assessment/ 851497

8 exp patient satisfaction/ 167147

9 ("clinical outcome" or "clinical outcomes" or expenditure* or expens* or benefi* or cost$2 or utilit* or assessment* or satisfaction or satisfied or effectiv* or success or survival* or recurrence* or progression or (quality adj2 life) or (therap* adj2 decision*) or "diagnostic accuracy" or precis* or impact or economic* or performance or financ*).ti,ab,kf. or de.fs. 12534662

10 6 or 7 or 8 or 9 13465340

11 exp patient/ 3000896

12 (patient* or diseas* or client* or injur* or disorder* or ill* or sick*).ti,ab,kf. 16659000

13 11 or 12 16852029

14 5 and 10 and 13 2091

15 (exp animal/ or exp invertebrate/ or nonhuman/ or animal experiment/ or animal tissue/ or animal model/ or exp plant/ or exp fungus/) not (exp human/ or human tissue/) 7711667

16 ((animal or animals or canine* or dog or dogs or feline or hamster* or lamb or lambs or mice or monkey or monkeys or mouse or murine or pig or pigs or piglet* or porcine or primate* or rabbit* or rats or rat or rodent* or sheep* or veterinar*) not (human* or patient*)).ti,kf,jx. 2729819

17 15 or 16 8031971

18 14 not 17 1524

19 (meta analysis or review or systematic review or case report).pt. or ("meta analysis" or "metaanalysis" or review or "case report" or "case study" or "case studies").ti. 3935606

20 18 not 19 1399

********************************************

**Cochrane Database**Search date: 2023/05/02

#1 ("7T" or "ultra high field" or uhf or "7 T" or "7 Tesla" or "seven tesla" or "7.0T" or "7.0Tesla" or "7.0 T" or "7.0 Tesla"):ti,ab,kw 1522

#2 (imaging or mri):ti,ab,kw 95975

#3 [mh "Magnetic Resonance Imaging"] 10793

#4 #1 AND (#2 OR #3) 193

#5 ("clinical outcome" or "clinical outcomes" or expenditure* or expens* or benefi* or cost or costs or utilit* or assessment* or satisfaction or satisfied or effectiv* or success or survival* or recurrence* or progression or (quality NEAR/2 life) or (therap* NEAR/1 decision*) or "diagnostic accuracy" or precis* or impact or economic* or performance or financ*):ti,ab,kw 1062314

#6 MeSH descriptor: [Treatment Outcome] explode all trees 179939

#7 [mh "Outcome Assessment, Health Care"] 189976

#8 [mh "Patient Satisfaction"] 15808

#9 #5 OR #6 OR #7 OR #8 1104884

#10 #4 AND #9 138

**In Trials 97**

********************************************

**Web of Science**Search date: 2023/05/02

| #1 | (TS=("7T" or "ultra high field" or uhf or "7 T" or "7 Tesla" or "seven tesla" or "7.0T" or "7.0Tesla" or "7.0 T" or "7.0 Tesla")) AND TS=(imaging or mri or mrt or tomograph*)  *Web of Science Core Collection Timespan = All years* | 9,027 |
| --- | --- | --- |
| #2 | TS=("clinical outcome*" or expenditure* or expens* or benefi* or cost? or utilit* or assessment* or satisfaction or satisfied or effectiv* or success or survival* or recurrence* or progression or (quality NEAR/3 life) or (therap* NEAR/2 decision*) or "diagnostic accuracy" or precis* or impact or economic* or performance or financ*)  *Web of Science Core Collection Timespan = All years* | 19,982,660 |
| #3 | TS=(patient* or diseas* or client* or injur* or disorder* or ill* or sick*)  *Web of Science Core Collection Timespan = All years* | 13,884,020 |
| #4 | #1 AND #2 AND #3 | 1,506 |
| #5 | TS=((animal or animals or canine* or dog or dogs or feline or hamster* or lamb or lambs or mice or monkey or monkeys or mouse or murine or pig or pigs or piglet* or porcine or primate* or rabbit* or rats or rat or rodent* or sheep* or veterinar*) not (human* or patient*))  *Web of Science Core Collection Timespan = All years* | 4,354,102 |
| #6 | #4 not #5 | 1,230 |
| #7 | TI=("meta analysis" or "metaanalysis" or review or "case report" or "case study" or "case studies")  *Web of Science Core Collection Timespan = All years* | 1,499,274 |
| #8 | #6 not #7 and Review Articles (Exclude – Document Types)  *Web of Science Core Collection Timespan = All years* | 1133 |

1. Borissov N, Haas Q, Minder B, et al. Reducing systematic review burden using Deduklick: a novel, automated, reliable, and explainable deduplication algorithm to foster medical research. Syst Rev. 2022;11(1):172. Published 2022 Aug 17. doi:10.1186/s13643-022-02045-9 [↑](#footnote-ref-1)
