# Supplementary material for: Assessment of the available evidence for the use of 7‐Tesla (T) magnetic resonance imaging (MRI) in neurological and musculoskeletal disorders, with comparison to 3‐T and 1.5‐T MRI: A systematic scoping review
Source: Eur J Neurol. 2024 Dec 15;32(1):e16557. doi: 10.1111/ene.16557 (PMC11647057; doi:10.1111/ene.16557)
Supplement: Supplementary file 2 — Appendix S2. [file ENE-32-e16557-s002.docx]

**References (publications included in data extraction)**

1. Absinta M, Sati P, Schindler MK, Reich DS, Fechner A. 3T MRI detection of 7T paramagnetic rims in multiple sclerosis lesions: A step toward the clinical application. *Multiple Sclerosis Journal*. 2017;23(3):79-80.

2. Chang G, Honig S, Liu Y, et al. 7 Tesla MRI of bone microarchitecture discriminates between women without and with fragility fractures who do not differ by bone mineral density. *Journal of bone and mineral metabolism*. 2015;33(3):285-93.

3. Theysohn JM, Kraff O, Maderwald S, et al. 7 tesla MRI of microbleeds and white matter lesions as seen in vascular dementia. *Journal of magnetic resonance imaging : JMRI*. 2011;33(4):782-91.

4. Obusez EC, Lowe M, Oh S-H, et al. 7T MR of intracranial pathology: Preliminary observations and comparisons to 3T and 1.5T. *NeuroImage*. 2018;168:459-476.

5. Leemans E, Cornelissen B, Sing MLC, et al. 7T versus 3T MR Angiography to Assess Unruptured Intracranial Aneurysms. *Journal of neuroimaging : official journal of the American Society of Neuroimaging*. 2020;30(6):779-785.

6. Tang MCY, Jaarsma-Coes MG, Ferreira TA, et al. A Comparison of 3 T and 7 T MRI for the Clinical Evaluation of Uveal Melanoma. *Journal of magnetic resonance imaging : JMRI*. 2021;

7. Harrison D, Allette Y, Cohen A, Choi S. An evaluation of the impact of MRI field strength and contrast delay on visualization of meningeal enhancement in multiple sclerosis. *Multiple Sclerosis Journal*. 2022;28(3 Supplement):267. 38th Congress of the European Committee for Treatment and Research in Multiple Sclerosis, ECTRIMS 2022. Amsterdam Netherlands. doi:<https://dx.doi.org/10.1177/13524585221123687>

8. Fartaria MJ, O'Brien K, Sorega A, et al. An Ultra-High Field Study of Cerebellar Pathology in Early Relapsing-Remitting Multiple Sclerosis Using MP2RAGE. *Investigative radiology*. 2017;52(5):265-273.

9. Stahl R, Krug R, Kelley DAC, et al. Assessment of cartilage-dedicated sequences at ultra-high-field MRI: comparison of imaging performance and diagnostic confidence between 3.0 and 7.0 T with respect to osteoarthritis-induced changes at the knee joint. *Skeletal radiology*. 2009;38(8):771-83.

10. Zbyn S, Schreiner M, Juras V, et al. Assessment of Low-Grade Focal Cartilage Lesions in the Knee With Sodium MRI at 7 T: Reproducibility and Short-Term, 6-Month Follow-up Data. *Investigative radiology*. 2020;55(7):430-437.

11. Friebe B, Richter M, Penzlin S, et al. Assessment of Low-Grade Meniscal and Cartilage Damage of the Knee at 7 T: A Comparison to 3 T Imaging With Arthroscopic Correlation. *Investigative radiology*. 2018;53(7):390-396.

12. Mistry N, Dixon J, Tallantyre E, et al. Central veins in brain lesions visualized with high-field magnetic resonance imaging: a pathologically specific diagnostic biomarker for inflammatory demyelination in the brain. *JAMA neurology*. 2013;70(5):623-8.

13. Schlamann M, Maderwald S, Becker W, et al. Cerebral cavernous hemangiomas at 7 Tesla: initial experience. *Academic radiology*. 2010;17(1):3-6.

14. De Santis S, Bastiani M, Droby A, et al. Characterizing Microstructural Tissue Properties in Multiple Sclerosis with Diffusion MRI at 7T and 3T: The Impact of the Experimental Design. *Neuroscience*. 2019;403:17-26.

15. Regnery S, Adeberg S, Dreher C, et al. Chemical exchange saturation transfer MRI serves as predictor of early progression in glioblastoma patients. *Oncotarget*. 2018;9(47):28772-28783.

16. de Graaf WL, Kilsdonk ID, Lopez-Soriano A, et al. Clinical application of multi-contrast 7-T MR imaging in multiple sclerosis: increased lesion detection compared to 3 T confined to grey matter. *European radiology*. 2013;23(2):528-40.

17. Beenakker JWM, Ferreira TA, Soemarwoto KP, et al. Clinical evaluation of ultra-high-field MRI for three-dimensional visualisation of tumour size in uveal melanoma patients, with direct relevance to treatment planning. *MAGNETIC RESONANCE MATERIALS IN PHYSICS BIOLOGY AND MEDICINE*. 2016;29(3):571-577.

18. Beisteiner R, Robinson S, Wurnig M, et al. Clinical fMRI: evidence for a 7T benefit over 3T. *NeuroImage*. 2011;57(3):1015-21.

19. van Egmond SL, Vonck BMD, Bluemink JJ, et al. Clinical value of (dedicated) 3 Tesla and 7 Tesla MRI for cT1 glottic carcinoma: A feasibility study. *Laryngoscope investigative otolaryngology*. 2019;4(1):95-101.

20. Cosottini M, Frosini D, Pesaresi I, et al. Comparison of 3T and 7T susceptibility-weighted angiography of the substantia nigra in diagnosing Parkinson disease. *AJNR American journal of neuroradiology*. 2015;36(3):461-6.

21. Oh BH, Moon HC, Baek HM, et al. Comparison of 7T and 3T MRI in patients with moyamoya disease. *Magnetic resonance imaging*. 2017;37:134-138.

22. Fechner A, Savatovsky J, Sadik JC, Roux P, Reich DS, Sati P. Comparison of central vein detection in MS at 1.5T, 3T and 7T. *Multiple Sclerosis Journal*. 2017;23(3):680.

23. Maranzano J, Dadar M, Rudko DA, et al. Comparison of cortical lesion frequency by type as detected by 3T and 7T multi-contrast MRI in patients with multiple sclerosis. *Multiple Sclerosis Journal*. 2018;24(2):615-616.

24. Springer E, Dymerska B, Cardoso PL, et al. Comparison of Routine Brain Imaging at 3 T and 7 T. *Investigative radiology*. 2016;51(8):469-82.

25. Springer E, Bohndorf K, Juras V, et al. Comparison of Routine Knee Magnetic Resonance Imaging at 3 T and 7 T. *Investigative radiology*. 2017;52(1):42-54.

26. Hangel G, Rausch I, Furtner J, et al. CORRESPONDENCE OF GLUTAMINE AND GLYCINE IMAGING BASED ON 7T MRSI TO AMINO ACID PET. *Neuro-Oncology*. 2022;24(Supplement 2):ii8. 17th Meeting of the European Association of Neuro-Oncology, EANO 2022. Vienna Austria. doi:<https://dx.doi.org/10.1093/neuonc/noac174>

27. Ni J, Auriel E, Martinez-Ramirez S, et al. Cortical localization of microbleeds in cerebral amyloid angiopathy: an ultra high-field 7T MRI study. *Journal of Alzheimer's disease : JAD*. 2015;43(4):1325-30.

28. Ghaznawi R, De Bresser J, Witkamp T, et al. Detection of small infarcts in the caudate nucleus on 7 tesla MRI: The smart-MR study. *Alzheimer's and Dementia*. 2017;13(7):P441-P442.

29. Morris L. Developing and Testing High-Resolution Functional MRI Methods and Relevance to Mood Disorders. *Biological Psychiatry*. 2022;91(9 Supplement):S31. Abstract Supplement. New Orleans United States. doi:<https://dx.doi.org/10.1016/j.biopsych.2022.02.096>

30. Radojewski P, Dobrocky T, Branca M, et al. Diagnosis of Small Unruptured Intracranial Aneurysms : Comparison of 7T versus 3T MRI. *Clinical neuroradiology*. 2023;doi:<https://dx.doi.org/10.1007/s00062-023-01282-2>

31. Moenninghoff C, Kraff O, Maderwald S, et al. Diffuse axonal injury at ultra-high field MRI. *PloS one*. 2015;10(3):e0122329.

32. Chalia M, Panda A, Brinkmann B, et al. Digging deep in 7T MRI: Potential epileptogenic and surgical lesions in epilepsy patients with nonlesional 3T MRI. *Epilepsia*. 2021;62:229-230.

33. Meissner J-E, Korzowski A, Regnery S, et al. Early response assessment of glioma patients to definitive chemoradiotherapy using chemical exchange saturation transfer imaging at 7 T. *Journal of magnetic resonance imaging : JMRI*. 2019;50(4):1268-1277.

34. Massacesi L, Vuolo L, Dewey B, Sati P, Reich DS. Efficiency of FLAIR*at 1.5T, 3T, and 7T for detecting perivenular lesions in multiple sclerosis (MS). *Multiple Sclerosis*. 2015;23(11):210.

35. Monninghoff C, Maderwald S, Theysohn JM, et al. Evaluation of intracranial aneurysms with 7 T versus 1.5 T time-of-flight MR angiography - initial experience. *RoFo : Fortschritte auf dem Gebiete der Rontgenstrahlen und der Nuklearmedizin*. 2009;181(1):16-23.

36. Cheng K, Duan Q, Hu J, et al. Evaluation of postcontrast images of intracranial tumors at 7T and 3T MRI: An intra-individual comparison study. *CNS neuroscience & therapeutics*. 2023;29(2):559-565. doi:<https://dx.doi.org/10.1111/cns.14036>

37. Duchin Y, Abosch A, Yacoub E, Sapiro G, Harel N. Feasibility of using ultra-high field (7 T) MRI for clinical surgical targeting. *PloS one*. 2012;7(5):e37328.

38. Barrett TF, Dyvorne HA, Padormo F, et al. First Application of 7-T Magnetic Resonance Imaging in Endoscopic Endonasal Surgery of Skull Base Tumors. *World neurosurgery*. 2017;103:600-610.

39. Kollia K, Maderwald S, Putzki N, et al. First clinical study on ultra-high-field MR imaging in patients with multiple sclerosis: comparison of 1.5T and 7T. *AJNR American journal of neuroradiology*. 2009;30(4):699-702.

40. de Rotte AAJ, Groenewegen A, Rutgers DR, et al. High resolution pituitary gland MRI at 7.0 tesla: a clinical evaluation in Cushing's disease. *European radiology*. 2016;26(1):271-7.

41. Hutter B-O, Altmeppen J, Kraff O, et al. Higher sensitivity for traumatic cerebral microbleeds at 7 T ultra-high field MRI: is it clinically significant for the acute state of the patients and later quality of life? *Therapeutic advances in neurological disorders*. 2020;13:1756286420911295.

42. Regnery S, Knowles BR, Paech D, et al. High-resolution FLAIR MRI at 7 Tesla for treatment planning in glioblastoma patients. *RADIOTHERAPY AND ONCOLOGY*. 2019;130:180-184.

43. Eisenhut F, Schmidt MA, Buchfelder M, Doerfler A, Schlaffer S-M. Improved Detection of Cavernous Sinus Invasion of Pituitary Macroadenomas with Ultra-High-Field 7 T MRI. *Life (Basel, Switzerland)*. 2022;13(1)doi:<https://dx.doi.org/10.3390/life13010049>

44. Abdel-Fahim R, Mistry N, Mougin O, et al. Improved detection of focal cortical lesions using 7T magnetisation transfer imaging in patients with multiple sclerosis. *Multiple sclerosis and related disorders*. 2014;3(2):258-65.

45. Fischer A, Maderwald S, Johst S, et al. Initial evaluation of non-contrast-enhanced magnetic resonance angiography in patients with peripheral arterial occlusive disease at 7 T. *Investigative radiology*. 2014;49(5):331-8.

46. Radojewski P, Seeck M, Vulliemoz S, Schindler K, Wiest R. Integration of high-resolution ultra-high-field 7T magnetic resonance imaging into clinical care of epilepsy patients: First results. *Epilepsia*. 2022;63(Supplement 2):209-210. 14th European Epilepsy Congress. Online. doi:<https://dx.doi.org/10.1111/epi.17388>

47. Santyr BG, Goubran M, Lau JC, et al. Investigation of hippocampal substructures in focal temporal lobe epilepsy with and without hippocampal sclerosis at 7T. *Journal of magnetic resonance imaging : JMRI*. 2017;45(5):1359-1370.

48. Kwan JY, Jeong SY, Van Gelderen P, et al. Iron accumulation in deep cortical layers accounts for MRI signal abnormalities in ALS: correlating 7 tesla MRI and pathology. *PloS one*. 2012;7(4):e35241.

49. Krusche-Mandl I, Schmitt B, Zak L, et al. Long-term results 8 years after autologous osteochondral transplantation: 7 T gagCEST and sodium magnetic resonance imaging with morphological and clinical correlation. *Osteoarthritis and cartilage*. 2012;20(5):357-363.

50. Kim J-M, Jeong H-J, Bae YJ, et al. Loss of substantia nigra hyperintensity on 7 Tesla MRI of Parkinson's disease, multiple system atrophy, and progressive supranuclear palsy. *Parkinsonism & related disorders*. 2016;26:47-54.

51. Sun K, Cui J, Wang B, et al. Magnetic resonance imaging of tuberous sclerosis complex with or without epilepsy at 7 T. *Neuroradiology*. 2018;60(8):785-794.

52. Wu Y, Agarwal S, Jones CK, et al. Measurement of arteriolar blood volume in brain tumors using MRI without exogenous contrast agent administration at 7T. *Journal of magnetic resonance imaging : JMRI*. 2016;44(5):1244-1255.

53. Lee Y, Kim K, Kang C, et al. Microvascular imaging of symptomatic and asymptomatic MCA steno-occlusive patients using ultra-high-field 7T MRI : A preliminary comparative study. *Cerebrovascular Diseases*. 2013;35:257.

54. Cong F, Zhuo Y, Yu S, et al. Noncontrast-enhanced time-resolved 4D dynamic intracranial MR angiography at 7T: A feasibility study. *Journal of magnetic resonance imaging : JMRI*. 2018;48(1):111-120.

55. Wrede KH, Matsushige T, Goericke SL, et al. Non-enhanced magnetic resonance imaging of unruptured intracranial aneurysms at 7 Tesla: Comparison with digital subtraction angiography. *European radiology*. 2017;27(1):354-364.

56. Wrede KH, Dammann P, Johst S, et al. Non-Enhanced MR Imaging of Cerebral Arteriovenous Malformations at 7 Tesla. *European radiology*. 2016;26(3):829-39.

57. Gascho D, Deininger-Czermak E, Zoelch N, et al. Noninvasive 7 tesla MRI of fatal craniocerebral gunshots - a glance into the future of radiologic wound ballistics. *Forensic science, medicine, and pathology*. 2020;16(4):595-604.

58. Yuan Y, Yu Y, Guo Y, et al. Noninvasive Delineation of Glioma Infiltration with Combined 7T Chemical Exchange Saturation Transfer Imaging and MR Spectroscopy: A Diagnostic Accuracy Study. *Metabolites*. 2022;12(10)doi:<https://dx.doi.org/10.3390/metabo12100901>

59. Marxreiter F, Lambrecht V, Mennecke A, et al. Parkinson's disease or multiple system atrophy: potential separation by quantitative susceptibility mapping. *Therapeutic advances in neurological disorders*. 2023;16:17562864221143834. doi:<https://dx.doi.org/10.1177/17562864221143834>

60. Wang ZI, Oh S-H, Lowe M, et al. Radiological and Clinical Value of 7T MRI for Evaluating 3T-Visible Lesions in Pharmacoresistant Focal Epilepsies. *Frontiers in neurology*. 2021;12:591586.

61. Veersema TJ, Ferrier CH, van Eijsden P, et al. Seven tesla MRI improves detection of focal cortical dysplasia in patients with refractory focal epilepsy. *Epilepsia open*. 2017;2(2):162-171.

62. Chou IJ, Lim S-Y, Tanasescu R, et al. Seven-Tesla Magnetization Transfer Imaging to Detect Multiple Sclerosis White Matter Lesions. *Journal of neuroimaging : official journal of the American Society of Neuroimaging*. 2018;28(2):183-190.

63. Zhang Y, Lv Y, You H, et al. Study of the hippocampal internal architecture in temporal lobe epilepsy using 7T and 3T MRI. *Seizure*. 2019;71:116-123.

64. van Laar PJ, Oterdoom DLM, Ter Horst GJ, et al. Surgical Accuracy of 3-Tesla Versus 7-Tesla Magnetic Resonance Imaging in Deep Brain Stimulation for Parkinson Disease. *World neurosurgery*. 2016;93:410-2.

65. Bian W, Hess CP, Chang SM, Nelson SJ, Lupo JM. Susceptibility-weighted MR imaging of radiation therapy-induced cerebral microbleeds in patients with glioma: a comparison between 3T and 7T. *Neuroradiology*. 2014;56(2):91-6.

66. Juras V, Schreiner M, Laurent D, et al. The comparison of the performance of 3T and 7T T2 mapping for untreated low-grade cartilage lesions. *Magnetic resonance imaging*. 2019;55:86-92.

67. Muthuraman M, Gonzalez-Escamilla G, Fleischer V, et al. The impact of field strength on structural network organization: A comparative study between 3T and 7T in multiple sclerosis patients. *Multiple Sclerosis Journal*. 2018;24(2):859-860.

68. Jakary A, Hess C, LaFontaine M, et al. The potential of 7T anatomical imaging for clinical assessment of contrast-enhancing and T2-hyperintense lesions in patients with glioma. *Neuro-Oncology*. 2017;19

69. Wiesmueller M, Meixner CR, Weber M, et al. Time-of-Flight Angiography in Ultra-High-Field 7 T MRI for the Evaluation of Peroneal Perforator Arteries Before Osseomyocutaneous Flap Surgery. *Investigative radiology*. 2023;58(3):216-222. doi:<https://dx.doi.org/10.1097/RLI.0000000000000926>

70. Rutland JW, Pawha P, Belani P, et al. Tumor T2 signal intensity and stalk angulation correlates with endocrine status in pituitary adenoma patients: a quantitative 7 tesla MRI study. *Neuroradiology*. 2020;62(4):473-482.

71. Salehi F, Nadeem IM, Kwan BYM, et al. Ultra-High Field 7-Tesla Magnetic Resonance Imaging and Electroencephalography Findings in Epilepsy. *Canadian Association of Radiologists journal = Journal l'Association canadienne des radiologistes*. 2021:8465371211031802.

72. Patel V, Liu C-SJ, Shiroishi MS, et al. Ultra-high field magnetic resonance imaging for localization of corticotropin-secreting pituitary adenomas. *Neuroradiology*. 2020;62(8):1051-1054.

73. Morris LS, Kundu P, Costi S, et al. Ultra-high field MRI reveals mood-related circuit disturbances in depression: a comparison between 3-Tesla and 7-Tesla. *Translational psychiatry*. 2019;9(1):94.

74. Germann C, Galley J, Falkowski AL, et al. Ultra-high resolution 3D MRI for chondrocalcinosis detection in the knee-a prospective diagnostic accuracy study comparing 7-tesla and 3-tesla MRI with CT. *European radiology*. 2021;31(12):9436-9445.

75. Eisenhut F, Schlaffer S-M, Hock S, et al. Ultra-High-Field 7 T Magnetic Resonance Imaging Including Dynamic and Static Contrast-Enhanced T1-Weighted Imaging Improves Detection of Secreting Pituitary Microadenomas. *Investigative radiology*. 2022;57(9):567-574. doi:<https://dx.doi.org/10.1097/RLI.0000000000000872>

76. Madai VI, von Samson-Himmelstjerna FC, Sandow N, et al. Ultrahigh-field MPRAGE Magnetic Resonance Angiography at 7.0 T in patients with cerebrovascular disease. *European journal of radiology*. 2015;84(12):2613-7.

77. Madai VI, von Samson-Himmelstjerna FC, Bauer M, et al. Ultrahigh-field MRI in human ischemic stroke--a 7 tesla study. *PloS one*. 2012;7(5):e37631.

78. Bartolini E, Cosottini M, Costagli M, et al. Ultra-High-Field Targeted Imaging of Focal Cortical Dysplasia: The Intracortical Black Line Sign in Type IIb. *AJNR American journal of neuroradiology*. 2019;40(12):2137-2142.

79. Sharma HK, Feldman R, Delman B, et al. Utility of 7 tesla MRI brain in 16 "MRI Negative" epilepsy patients and their surgical outcomes. *Epilepsy & behavior reports*. 2021;15:100424.

80. Rutland JW, Delman BN, Feldman RE, et al. Utility of 7 Tesla MRI for Preoperative Planning of Endoscopic Endonasal Surgery for Pituitary Adenomas. *Journal of neurological surgery Part B, Skull base*. 2021;82(3):303-312.

81. Wang I, Oh S, Blumcke I, et al. Value of 7T MRI and post-processing in patients with nonlesional 3T MRI undergoing epilepsy presurgical evaluation. *Epilepsia*. 2020;61(11):2509-2520.

82. Isaacs BR, Heijmans M, Kuijf ML, et al. Variability in subthalamic nucleus targeting for deep brain stimulation with 3 and 7 Tesla magnetic resonance imaging. *NeuroImage Clinical*. 2021;32:102829.

83. van der Jagt MA, Brink WM, Versluis MJ, et al. Visualization of human inner ear anatomy with high-resolution MR imaging at 7T: initial clinical assessment. *AJNR American journal of neuroradiology*. 2015;36(2):378-83.
